# Immunological Processes Driving IgE Sensitisation and Disease Development in Males and Females

**DOI:** 10.3390/ijms19061554

**Published:** 2018-05-23

**Authors:** Jonatan Leffler, Philip A. Stumbles, Deborah H. Strickland

**Affiliations:** 1 Telethon Kids Institute, The University of Western Australia, 100 Roberts Rd, Subiaco, WA 6008, Australia; p.stumbles@murdoch.edu.au (P.A.S.); Deb.Strickland@telethonkids.org.au (D.H.S.); 2 School of Paediatrics and Child Health, The University of Western Australia, Subiaco, WA 6008, Australia; 3 School of Veterinary and Life Sciences, Murdoch University, Murdoch, WA 6150, Australia

**Keywords:** IgE sensitisation, respiratory sensitisation, sex hormones, allergic asthma, innate immunity, adaptive immunity

## Abstract

IgE sensitisation has increased significantly over the last decades and is a crucial factor in the development of allergic diseases. IgE antibodies are produced by B cells through the process of antigen presentation by dendritic cells, subsequent differentiation of CD4^+^ Th2 cells, and class switching in B cells. However, many of the factors regulating these processes remain unclear. These processes affect males and females differently, resulting in a significantly higher prevalence of IgE sensitisation in males compared to females from an early age. Before the onset of puberty, this increased prevalence of IgE sensitisation is also associated with a higher prevalence of clinical symptoms in males; however, after puberty, females experience a surge in the incidence of allergic symptoms. This is particularly apparent in allergic asthma, but also in other allergic diseases such as food and contact allergies. This has been partly attributed to the pro- versus anti-allergic effects of female versus male sex hormones; however, it remains unclear how the expression of sex hormones translates IgE sensitisation into clinical symptoms. In this review, we describe the recent epidemiological findings on IgE sensitisation in male and females and discuss recent mechanistic studies casting further light on how the expression of sex hormones may influence the innate and adaptive immune system at mucosal surfaces and how sex hormones may be involved in translating IgE sensitisation into clinical manifestations.

## 1. Introduction

It has been known for some time that sex hormones influence the function and activity of the immune system and likely contribute to the sex-based differences in immune-mediated disease responses. These effects are presumably a result of the different evolutionary needs of males and females. In general, these effects have resulted in adult females inducing a more efficient humoral-based immune response upon pathogen challenge, whereas males tend to mount a less efficient immune response [1,2]. The main female sex hormones include estrogen and progesterone, whereas the main male sex hormone is testosterone. These hormones mediate their effects through binding to receptors expressed on the surface of a wide array of cell types, to either directly induce gene expression or activate signalling cascades from the cell surface. Estrogen mediates its effects through three receptors, i.e., estrogen receptors α and β and G-protein-coupled receptor 1 [3]. Progesterone mediates its effects through two isoforms (A and B) of the progesterone receptor [4], and testosterone operates through the androgen receptor NR3C4 [5]. Different receptors may have different downstream effects and may also exists in several isoforms. These receptors are expressed by several types of immune cells, including innate antigen-presenting and adaptive immune cells [6,7].

The generalised effects of male and female sex hormones are such that estrogen and progesterone tend to enhance, whereas testosterone acts to inhibit immune responses [1,2]. This differential effect has been linked to comparative differences in the prevalence of several diseases in male and females, including parasite infections and a range of non-communicable diseases [8]. Parasitic infections are mainly prevented through humoral immunity and thus appear less common in females because of their enhanced humoral immune response. The immune-enhancing effects of female sex hormones also increase the susceptibility of females to several autoimmune diseases, such as multiple sclerosis, systemic lupus erythematosus, and rheumatoid arthritis, which are all significantly more common in females than in males [9]. A differential prevalence amongst men and women is also evident for allergic diseases such as allergic asthma, atopic dermatitis, and food allergies, which in general appear to initially affect young males more than females; however, in post-pubertal females, allergic disease incidence rates increase to become equivalent to or exceed those in post-pubertal males [10,11,12,13,14,15].

Our group’s research interests have primarily focussed on respiratory sensitisation to airborne allergens and subsequent development of allergic asthma. Thus, for the purposes of this review, we will focus on IgE sensitisation and progression to symptomatic disease within the respiratory tract and review the current knowledge of how sex hormones influence this process.

## 2. Atopy and IgE Sensitisation

The inception of the pathological processes that lead to the development of allergic disease(s) usually occurs much earlier than the initial display of the clinical symptoms. Patients often develop a condition known as atopy, which is defined by elevated levels of allergen-specific IgE production. However, while IgE sensitisation is a necessary precursor to the development of allergy, it does not necessarily dictate that clinical allergic disease will develop in an individual. Approximately 50% of the population in western countries is positive for allergen-specific IgE antibodies as determined by specific serum IgE measurements or skin prick test (SPT) reactivity [16,17]. However, only approximately 50% of IgE-sensitised individuals (or 20–25% of the population) suffer from an allergic disease [18]. This suggests that there are specific mechanisms that can potentially drive or provide protection against the translation of atopic sensitisation to clinical symptomatic disease. Although the mechanisms underlying the transition from IgE sensitisation into allergic disease are poorly understood, the genetic background together with allergen and environmental exposures play a large role in both IgE sensitisation and its translation to symptomatic disease [19]. Further, the risk of clinical disease also increases with the level of sensitisation determined by IgE serum levels and SPT wheel size [20,21]. In addition, several studies have shown that growing up in a farming environment or other microbially rich environments such as with indoor pets appears protective, even in genetically similar populations [22,23,24]. IgE sensitisation is usually not specific for a single allergen but, instead, sensitisation in each individual occurs to a range of allergens encountered through various routes over time [25]. Consequently, it is common that an allergic individual suffers from multiple allergic diseases, often in a sequential pattern of development termed the “atopic march”, where IgE-sensitisation and development of allergic disease follow a defined pattern, initially affecting the skin and gut, and later the airways [26,27].

### IgE Sensitisation in Male and Females

**Figure 1:**
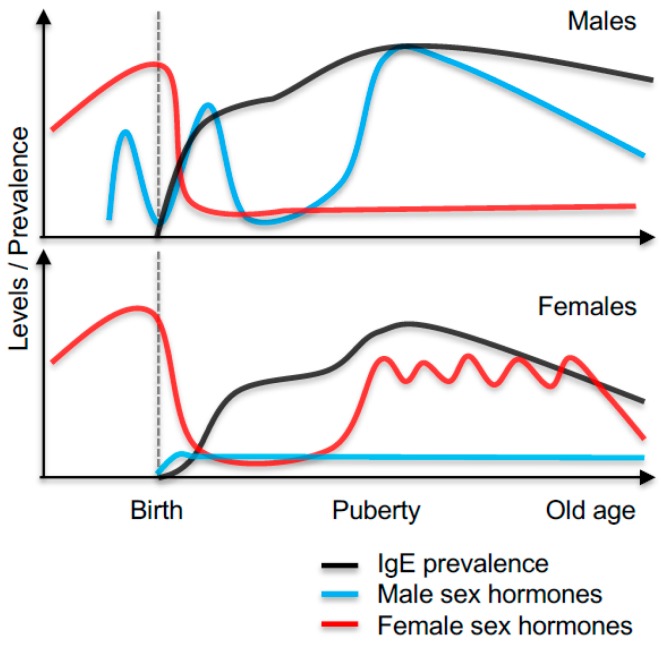
Illustration of the prevalence of IgE sensitisation and levels of male and female sex hormones throughout life in male and females.

Allergen-specific IgE sensitisation can occur as early as during the first year of life, and some reports propose that sensitisation already occurs in utero, although this is controversial [28,29]. Several studies suggest that, as both children and adults, males have higher total and allergen-specific IgE levels than females [16,30]. In high-risk cohort studies, differences in circulating IgE levels and development of a positive skin prick test (SPT^+^) first appear at a very young age [31]. Before the age of one, the rates of sensitisation are significantly higher in males (33% SPT^+^) than in females (22% SPT^+^, [31]), with higher levels of total IgE in SPT^+^ boys compared to SPT^+^ girls [31,32,33]. At this age, total IgE levels appear to be under a strong genetic influence and may not predict total IgE levels in the same individual later in childhood [34,35]. Although the rates of IgE sensitisation increase with age in females, the increased levels of IgE and the prevalence of sensitisation in males remain into early adolescence [32,35,36,37]. At this age, 60% of the population is sensitised to an allergen [38]. Although not all studies on IgE levels in children observe a male predominance [33,39], the data do suggest that very young males are more prone than females to develop IgE sensitisation following allergen exposure [31]. The mechanism for these differences are not clear but could be attributed to genetic differences (described in more detail below) or to differential sex hormone exposures in utero or postnatally. It has been proposed that the surge in the rates of sensitisation among girls following puberty is most likely due to changes in sex hormone levels at this time [40,41]. The prevalence of IgE sensitisation in males and females, as well as hormone exposures throughout life, is illustrated in Figure 1.

After puberty, the total IgE and specific allergen IgE levels appear to remain higher or comparable in men compared to women [16,30,42,43,44]. In later adulthood, the IgE levels appear in general to decrease in both sexes [45,46]. In addition to changes in the IgE levels throughout life, the levels of IgE also vary with environmental exposures during the pollen season and are influenced by the menstrual cycle in females, suggesting a role for female sex hormones in regulating IgE levels [46,47].

## 3. Respiratory Allergic Sensitisation: The Mechanisms in Rodents and Human

**Figure 2:**
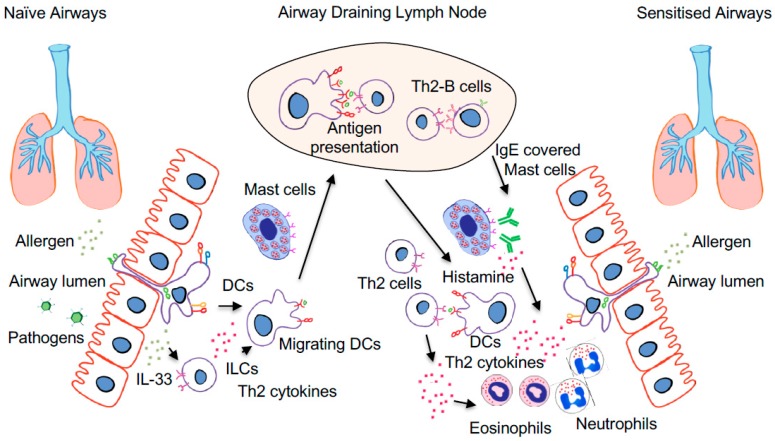
The process of respiratory IgE sensitisation is initiated by allergen inhalation in naïve airways (**left**) followed by antigen uptake by dendritic cells (DCs) lining the airway epithelium. Depending on the local microenvironment, pro/anti-inflammatory factors are released by the airway epithelium or innate lymphoid cells (ILC) that activate allergen-carrying DCs that migrate to the airway draining lymph nodes. Here, allergen-carrying DCs present the allergen to the adaptive immune system. In the case of IgE sensitisation, the presentation of an allergen to naïve CD4^+^ T cells induces Th2 differentiation and formation of Th2 effector cells that migrate back to the airways. In addition, the Th2 response also leads to the production of allergen-specific IgE by B cells that bind to mast cells and DCs in the airways. Upon allergen re-exposure (**right**), the allergen-induced IgE crosslinking on mast cells leads to histamine release and infiltration of innate immune cells such as eosinophils and neutrophils. In addition, antigen uptake and local presentation by DCs also result in Th2 cell activation, which further fuels the infiltration of immune cells, leading to symptomatic allergic airway disease.

**Figure 3:**
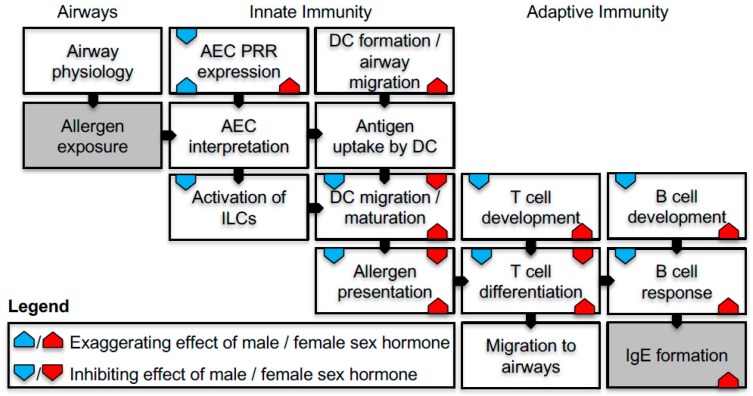
Schematic illustration of the processes involved in IgE sensitisation and a summary of how male (blue) and female (red) sex hormones influence each process. Exaggerating effects (triagle upward pointing symbol), inhibiting effects (downward pointing symbol) or both exaggerating and inhibiting effects depending on the study (triangle up and down), as discussed in this review. Figure specific abbreviations; PRR: pathogen recognition receptor, ILC: innate lymphoid cells, AEC airway epithelial cells:

Given the change in the prevalence and rates of IgE sensitisation in young versus adolescent males and females, it has been suggested that sex hormones play an important part in the immunological mechanisms of IgE sensitisation. In the sections below, we will describe what is currently known of the immunological processes involved in respiratory sensitisation, with a focus on antigen uptake and presentation followed by cellular and humoral adaptive responses and on how these processes are influenced by sex hormones. A simplified overview of the immunological processes involved in sensitisation is illustrated in Figure 2, and the effects of sex hormones on the sensitisation process are summarised in Figure 3.

### 3.1. Aeroallergen Uptake and Presentation by Airway Dendritic Cells

Respiratory IgE sensitisation is initiated through the exposure, inhalation, and uptake of allergens by specialised antigen-capture and -presenting cells termed dendritic cells (DCs) that reside in the airway mucosa and lung parenchyma [48,49,50]. These DCs are distributed in a network through the airway epithelial layer where they monitor the inhaled substances [51]. In the airways, there are three types of resident DCs which have most recently been defined as conventional DC1 (cDC1), cDC2, and plasmacytoid DC (pDC). The cDC1 subset expresses BATF3 and includes what were historically named CD103^+^ cDC in mouse, and BDCA3^+^ or, alternatively, CD141^+^ cDC in humans [52]. The cDC2 subset expresses IRF4 and includes what were previously termed CD11b^+^ cDC in mice and BDCA1^+^ cDC or, alternatively, CD1c^+^ cDC in humans [52]. Within the cDC1 subset, there are both CD103-positive and -negative subsets, and, within the cDC2 subset, not all cells express CD11b [52]. There is also a population of monocyte-derived DC (moDC) in mouse airways, although this population appears transient and mainly infiltrates the airways in response to inflammatory stimuli [53]. This subset is mainly involved in the execution of Th2 effector functions and in the production of pro-inflammatory cytokines in sensitised individuals [54,55]. Despite years of intense research, it is still unclear if a specific DC subset is involved in inducing the underlying Th2-biased immunity that leads to IgE sensitisation or if, instead, the airway microenvironment influences the DC activation status and capacity to induce different T cell response types [56,57]. The antigen load and relative cell numbers and ratios of DC subsets also play a central role in balancing between sensitisation and tolerance induction [56,58,59,60]. This is exemplified by observations suggesting that immature DCs preferentially induce tolerance, and a high-dose allergen may also favour a non-Th2-dominated response, as reviewed [61,62].

Several studies on the development of Th2-mediated IgE sensitisation in mice, however, point towards a major role of IRF4^+^ cDC2 cells [54,63,64], although Th2 sensitisation may also be induced through deficient allergen presentation by cDC1 [65,66] or pDCs [67,68]. BATF3^+^ cDC1 are central in inducing and maintaining tolerance to self-antigens through the presentation of apoptotic material and are also involved in inducing CD8^+^ T cells [69]. Plasmacytoid DCs are mainly involved in inducing type-1 interferon responses upon viral infections [70]; however, in the context of Th2 sensitisation, they have also been shown to reduce allergen-specific T cell responses [67,68].

### 3.2. The Influence of Sex Hormones on DC Function

Several studies have shown that DCs are highly influenced by sex hormones (as reviewed recently [71]). The effects of sex hormones on DCs range from those from early observations showing that DCs from females appears more efficient in inducing T cell proliferation [72], to those of more recent observations showing that, during DC development, early CD11c^+^/CD11b^in^/Ly-6C^−^ DC precursors are dependent on estrogen signalling through estrogen receptor alpha (ERα) and GM-CSF to develop into IRF4^+^ cDC2 [73,74,75]. Estrogen also influences MHC-II expression in DCs and their capacity to produce cytokines such as IL-6 and IL-12 [73], stimulation of CD4^+^ T cells [75,76], and DC migration [77,78]. If DCs mature in the absence of estrogen or ERα, a pre-DC-like CD11b^hi^/Ly-6C^hi^/MHC-II^lo^ subset, similar to what was recently described [79], become the dominant subset [76]. Some reports suggest that progesterone inhibits DC activation through reduction of MHC-II and CD86 expression and reduces the ability of DCs to activate CD4^+^ T cell proliferation. [80,81]. Of note is that the hormone dose appears crucial, given that high progesterone doses may also be able to enhance the differentiation of DCs from the decidua in vitro [82]. Similar effects have also been ascribed to testosterone, including the inhibition of pro-inflammatory cytokines by DCs [83]. Hence, the removal of testosterone through castration results in the upregulation of MHC-II expression in DCs as well as in the induction of a stronger immune response to immunisation compared to untreated male mice [84]. In pDCs, estrogen may influence the ability to produce IFN-α in response to toll-like receptors (TLR) ligands [85], and there are reports suggesting that estrogen may also impact the development of pDCs by influencing IRF5 expression [86,87]. Plasmacytoid DCs from infant males also appear less able to induce an immune response to TLR stimulation compared to female pDCs [88]. Given that DCs have a central role in orchestrating the immune responses, the influence of sex hormones on DC activation may have large implications into the subsequent immune responses, such as increased IgE sensitisation.

### 3.3. The Influence of Sex Hormones on Innate Immune Sensing in the Airways

In the airways, the mucosal microenvironment is constantly monitored by the interactions between resident airway epithelial cells and migrating allergen-sampling cDCs [89,90]. Innate immune receptors and enzyme cascades such as TLR and the complement system help the airway epithelial cells detect danger signals in the mucosal microenvironment and facilitate the interpretation of the allergen exposure context. Low rates of complement activation appear to be crucial in induction of immune tolerance [91]; however, complement activation in sensitised individuals may contribute to airway inflammation [92]. The expression of TLR4 in the airway epithelial cells has been shown to be essential for the induction of Th2 immunity in response to house dust mite exposure in mice [93] and may also be involved in the sensitisation to other allergens such as cockroach and ovalbumin [94,95]. Allergen exposure induces the airway epithelial cells to release the cytokines TSLP, IL-33, and IL-25. TSLP induces DC maturation and activation, plays a central role in Th2 immunity [96,97,98], and acts together with IL-33 to activate other innate immune cells, such as ILC2 that initiates and amplifies the production of Th2 cytokines such as IL-13 in the airways [99,100]. These further facilitate DC migration towards the airway draining lymph nodes [101]. Curiously, a continuous activation of TLR4 may also be involved in the protection against sensitisation mediated through endotoxin exposure [102] and may be a central pathway of the hygiene hypothesis [103,104]. Recent findings also suggest that IL-33 is involved in the induction of Th1 responses in mice and human [105,106], hinting that these processes may be more complex than currently depicted.

There are several reports suggesting that TLRs are differently expressed in male and females, particularly TLR7 which is expressed by genes on the X chromosome and in some cases has been described as able to avoid chromosome silencing [107]. TLR7 is stimulated by viral ssRNA, which appears to induce a stronger anti-viral response in females than in males [108]. In particular, ssRNA stimulation results in IFN-α production by pDCs, which may be related to elevated levels of IRF5 in females compared to males [86]. In contrast, TLR4 expression appears to be inhibited by testosterone in mouse macrophages [109]. However, this effect is most likely more complex, given that in vivo TLR4 expression is slightly elevated in male mice, resulting in a stronger cytokine (IL-6) response upon LPS stimulation of male compared to female macrophages [110]. In addition, TLR4 expression in macrophages also appears to be dependent on estrogen, and elevated estrogen levels increase TLR4 expression [111]. Complement proteins and complement receptors are also under the influence of sex hormones in some tissues such as the liver, where the majority of complement proteins are produced [112,113,114]. Whether this also holds true in the airways and if this further relates to clinical outcomes remains to be elucidated. Together, the interpretation of these findings is that sex hormones may influence our genetic predisposition to how a potential danger signal is sensed and translated into immunological responses.

### 3.4. Sex Hormone Influences on the Induction of CD4^+^ T Cell-Mediated Immune Responses

CD4^+^ T helper (Th) cells are the major T cell subset driving allergic sensitisation and are generally further subdivided into Th1, Th2, Th17, Th9, and T regulatory (Treg) subsets. The Th1 subset primarily produces IFN-γ and is involved in antimicrobial defences through IgG sensitisation and macrophage-mediated cytotoxicity. The Th2 subset is characterised by the production of IL-4 and drives humoral responses and class switching to IgE, which makes them the dominant Th cell subset in allergic sensitisation. Th17 cells play an important role in chronic inflammation through the production of IL17A and IL-21 [115], while Th9 cells produce IL-9 and are involved in a range of inflammatory diseases including allergic diseases [116]. In contrast to the above subsets that promote inflammation, the Treg subset plays a pivotal role in regulating immune responses, including the ability to inhibit the clinical symptoms of allergic airways disease in animal models [117] and humans, as reviewed [118]. The role of DCs in the primary activation of Th2 cells in allergic disease has received much attention in the literature; however, many aspects still remain unclear. Once aeroallergen-loaded DCs migrate to the airway draining lymph nodes, they interact with and activate allergen-specific naïve CD4^+^ cells which, in the case of IgE sensitization, initiate the production of IL-4, IL-5, and IL-9 and differentiate into Th2 cells [54]. This process is dependent on several factors, including DC cytokine production, antigen and DC load, microenvironmental influence, as well as DC subset. Although the exact timing and order of formation remains unclear [119,120], the ensuing Th2 response results in the activation of IL-4- and IL-21-producing T follicular helper (T_FH_) cells that initiate and support IgE production by B cells in germinal centres [121,122]. This effect appears to be reciprocal, with B cells also playing an important role in priming T follicular helper (T_FH_) cells [119]. Once activated, allergen-specific Th2 cells express homing receptors that guide migration from the lymph nodes back to the airways, where they then receive additional local microenvironmental signals to produce pro-allergic cytokines such as IL-5 in response to allergen re-challenge, resulting in the recruitment of eosinophils and other inflammatory cells into the airways.

### 3.5. T Cell Development and Activation Are Influenced by Sex Hormones

From birth, females display a higher proportion of CD4^+^ T cells than boys, and this difference also remains throughout adulthood [123,124,125]. This may in part be due to the inhibitory effects that testosterone has on T cell output from the thymus [126]. Estrogen is also known to be involved in T cell development, acting mainly through ERα [127,128], potentially reducing IL-2 expression [129] which is crucial for T cell activation and Treg development [130]. Both estrogen and testosterone influence T cell activation and the Th1/Th2 balance [131,132,133,134]. This appears to be dependent on estrogen concentrations, with a low dose supporting induction of Th1 and luteal/pregnancy-equivalent levels promoting Th2 induction by upregulating IL-4 and GATA-3 expression in CD4^+^ T cells after CD3/28 stimulation [135]. Testosterone also appears to dampen both Th1 activation, through the inhibition of IL-12-activated STAT4 [136], and Th2 activation, leading to a reduced ability to clear parasite infections [137]. IL-4 producing T_FH_ cells are also influenced by sex hormones, and their proportions correlate with estrogen levels during pregnancy [138]. The exposure to estrogen also increases IL-21 expression in CD4^+^ T cells and may enhance B cell proliferation and induction of Th17 cells [139]. One possible mechanism for how estrogen influences T_FH_ cells is through blockage of PPARγ signalling, which is a master regulator of T_FH_ and other effector T cells [140]. Activation of PPARγ appears to prevent T_FH_, as PPARγ^−/−^ mice have more T_FH_ cells compared to wild-type (WT) mice [141], suggesting that PPARγ is necessary in females to prevent and control T_FH_ induction and the production of antibodies.

As described above, Treg play a central role in preventing the expression of allergic symptoms and have been shown to be present in reduced numbers in the airways of allergic individuals compared to healthy controls [118]. In humans, Tregs are central for maintaining immunological tolerance in the foetus, and Treg levels also increase during the course of pregnancy [142]. This is largely controlled by female sex hormones levels, which result in increased proportions of Tregs in females compared to males [143] and to fluctuations of the levels of Tregs and other T cell subsets during the menstrual cycle [144]. In mice, however, ovariectomy curiously increased the proportion of Tregs in the CD4^+^ population in the spleen and lymph nodes as well as reduced the levels of IL-4, IL-5, and IL-13 in response to ovalbumin challenge [145]. However, whether this was an actual decrease in Treg numbers or simply a contraction of FoxP3^−^/CD4^+^ cells remains unclear. In another study, estrogen treatment increased, instead, CD4^+^ Treg numbers and *FoxP3* gene expression in vivo and enhanced FoxP3 protein expression after activation of CD25^−^/CD4^+^ T cells in vitro [146]. Notably, the Treg-defining transcription factor FoxP3 is also expressed on the X chromosome [147].

### 3.6. Sex Hormone Influences on B Cell Responses and IgE Sensitisation

B cells are mainly found in lymphoid tissues and form germinal centres together with CD4^+^ T_FH_ cells and follicular dendritic cells. Germinal centres support B cell survival, class switching, B cell receptor maturation, and induction of B cell memory. B cells are activated and differentiate into antibody-producing plasma cells through the help of CD4^+^ T_FH_ in a CD40L- and IL-21-dependent manner. Expression of IL-4 by T_FH_ induces B cell class switching into IgE; however, IgE-expressing B cells are extremely rare and appear to quickly differentiate into plasma cells and disappear out of the germinal centres [148,149]. However, long-lived IgE-positive B cells likely reside in the bone marrow and spleen [150], and IgE-based allergies can be transferred by bone marrow transplants [151]. Recently, B and T cell interactions outside lymphoid tissues, such as in the lung, have been observed; however, these interactions do not appear dependent on classical T_FH_ cells [152]. Given the potency of IgE, some studies suggest that IgE-producing B cells may be generated de novo from IgG^+^ memory B cells upon appropriate stimulation and context [153]. Interestingly, IL-21 appears to induce apoptosis in IgE-producing B cells [154], possibly providing a regulatory mechanism ensuring transient IgE responses in most individuals. Some reports in mice suggest that, once memory B cells are established, CD4^+^ cells are no longer required for secondary allergen recall responses and IgE maturation [155]. This also appears consistent with findings in allergic patients with HIV and depleted CD4^+^ T cells [156]. 

It has been known for some time in humans that males and females differ in the serum concentrations of circulating antibodies, particularly IgM [157], while estrogen and estrogenic compounds are able to increase IgE production in mouse spleen [158], possibly through ERβ signalling [127]. In humans, females display higher numbers of several B cell subsets compared to males [159,160], and these B cells also differ in their gene expression profiles between males and females [161]. Estrogen has been shown to negatively impact the ability to induce B cell tolerance [162] and promote B cell expansion and a lower threshold of B cell activation [163,164,165]. In contrast, estrogen induced IL-10-producing regulatory B cells in an experimental autoimmune encephalitis model [166]. These differential effects may be due to estrogen dose or model-specific effects in the respective studies. Progesterone also influences B cell function [167] and interestingly increases the proportion of IL-10-producing B regulatory cells [168] which are able to prevent allergic IgE-mediated mast cell responses in the airways [169]. Testosterone has been shown to directly inhibit antibody production [170] and prevent B cell maturation [171,172]. These effects and an early surge in testosterone in males following birth may provide an explanation why young boys display lower proportions of mature B cells [173], which has also been associated with an increased risk of developing allergic disease and IgE sensitization, compared to pre-puberty girls [174].

## 4. After Sensitisation—Translation into Clinical Allergic Disease

As mentioned above, the majority of epidemiological data suggest that males have higher IgE levels and prevalence of IgE-sensitisation at young and adult ages than females [16,37,43,175,176]. This can be linked to the increased risk of young males to develop IgE-associated clinical manifestations, such as asthma and wheeze [32]. Interestingly, the risk associated with clinical manifestations changes with age and at the age around puberty, and the risk of clinical manifestations associated with IgE levels appears equal for boys and girls [177]. After puberty, and although males still display higher prevalence of SPT^+^ as well as higher IgE levels compared to females [178], females are either at equal or at an increased risk of developing allergic diseases [18,175,179]. Indeed, a UK study that surveyed the presentations to medical clinics after puberty reported that females presented more often with asthma-, rhinitis-, and eczema-associated symptoms compared to boys [180]. Similarly, a US study showed that females appear to have higher rates of physician-diagnosed, although not self-reported, respiratory allergy than males [181]. Together, this suggests that, after puberty, females may be less efficient in preventing the translation of IgE sensitisation into clinical allergic diseases such as allergic asthma.

### Sex Differences in Asthma Pathology

Before puberty, asthma mainly affects males, whereas, after puberty, asthma transforms into a disease that affects more females, particularly in the presence of other allergic comorbidities such as rhinitis [182]. In children, the male gender is an independent risk factor for developing wheeze at the age of six [183], whereas, in adolescence, testosterone may instead protect against asthma. At the same time, female sex hormones instead contribute to airway hyper-responsiveness (AHR) [184]. In adults, asthma generally results in more hospitalisations for females compared to males, and females also appear more asthma-prone at particular stages of the menstrual cycle and during pregnancy [185], potentially in relation to the sex of the foetus [186]. Some of this skewed prevalence can be explained by female physiology. Estrogen signalling is involved in airway development [187], resulting in smaller airways in females, which possibly also makes obese females more prone to develop asthma than obese boys [188]. Female sex hormones also induce airway mucus production [189] and influence cilia movements [190], which may both contribute to asthma pathology in females.

In addition to these physiological factors, the influence of sex hormones on the immune system likely plays a large role in the skewed asthma susceptibility in male and females, as reviewed [185]. Female sex hormones also appear to be involved in aspects of the sensitisation process that are independent of IgE production, given that ovariectomy prior to sensitisation subsequently reduced the recall response in female mice, whilst the IgE levels remained unchanged [191]. Several experimental studies have also reported that female rodents in general develop stronger immune responses to sensitization, with higher allergen-specific IgE titres [192,193,194]. However, this does not appear to be reflected in human epidemiological data, as discussed above. Along these findings, several experimental studies have shown that either ovariectomy [195] or the use of tamoxifen [196] as an estrogen antagonist reduced eosinophil airway infiltration in response to ovalbumin challenge in mice and rats as well as an altered, less Th2-dominated, cytokine response in the airways [197]. These alterations could usually be restored by supplementation of estrogen or progesterone [195,197]. Mice treated with the estrogen antagonist tamoxifen also displayed a reduced ability of CD4^+^ T cell to produce Th2 cytokines in response to ovalbumin stimulation, further supporting a role for female sex hormones in promoting Th2 responses in vivo [196]. Female sex hormones also appear to have fast-acting effects on the airways, and, when progesterone was applied to the trachea of sensitised mice just prior to allergen challenge, an increased eosinophil infiltration and AHR were observed [198]. However, not all experimental studies support the existence of asthma-prone effects of estrogen and have instead shown estrogen administration to decrease ovalbumin-induced AHR, production of IL-5 and IL-13, as well as eosinophil recruitment. This was further associated with an increase in CD4^+^/FoxP3^+^ cells in an IL-10- and estrogen receptor GPR1-dependent manner [199]. Estrogen also reduced carbachol-induced and IgE-enhanced airway constriction of mice airways in vitro [200] and also reduced methacholine-induced AHR in male mice repeatedly exposed to ovalbumin [201]. In the context of asthma, testosterone has been associated with a reduction of the allergic responses in mice [202] and has also been suggested as a potential asthma treatment in humans [203]. Recently, a novel mechanism for how testosterone reduced asthma symptoms in an experimental model was described to operate through the inhibition of the development and migration of innate lymphoid cells into the airways [204].

In conclusion, it remains unclear how sex hormones directly influence the process of sensitisation in young, adolescent, and adults, as apparent from the discrepancy between experimental and epidemiological studies. There is, however, a clear role for sex hormones in modifying the symptomatic allergic recall responses following sensitization, as supported by both experimental and epidemiological studies. Together, these studies suggest that female sex hormones in general appear to enhance the immunological recall responses, likely leading to exaggerated disease, whereas testosterone dampens the same responses. These effects increase the risk of allergic diseases in adult females, although, paradoxically, females in general appear to display both lower levels of IgE and prevalence of sensitisation compared to males.

## 5. Sex Hormones and Impact on Therapies for Allergic Sensitisation

Although sex differences clearly modulate the risk of translating IgE sensitisation into clinical manifestations in the form of eczema or allergic asthma, relatively little is understood of how sex hormones contribute to the process of sensitisation. The majority of experimental research has instead focused on the effector phase and recall responses following sensitisation. This is unfortunate, given that these natural differences in the risk of sensitisation provide a unique opportunity to study and better understand the immunological processes involved. Further, the realisation that fluctuations in sex hormones may impact disease pathology is central to the administration of successful treatments by modifying the treatments during high-risk periods.

The mechanisms by which the sex hormones influence IgE sensitisation and allergic recall responses create opportunities to improve disease control by stabilizing hormone levels through the use of oral contraceptives, and studies support the intake of oral contraceptives in order to modulate asthma responses [205]. However, such strategies will need refinement, since an increased risk of asthma or wheeze in non-asthmatics was also observed [206]. Furthermore, hormonal replacement in older women has also been associated with an increased risk of asthma [207]. Estrogen modulators such as tamoxifen also have the potential to reduce asthma symptoms, as demonstrated in preclinical models of neutrophilic asthma [208]. Other potential therapies involve the ERα antagonist fulvestrant (ICI182.780), which has demonstrated some clinical effects in female lupus patients by preventing estrogen–ERα signalling in pDCs [209] and may also be beneficial in a subset of asthma patients. The development of specific estrogen analogues [210] targeting specific immunological effects without interfering with the reproductive effects will likely be possible in the future and warrants further investigation.
